# Variety of Neuropsychological Deficits and Clinical Rehabilitation Course After Surgical Removal of Cerebral Meningioma Under Neuropsychological Therapy

**DOI:** 10.3390/brainsci16040416

**Published:** 2026-04-15

**Authors:** Stefanie Auer, Peter Gugel, Natalie Gdynia, Andreas Gratzer, Ingo Haase, Hans-Jürgen Gdynia

**Affiliations:** 1 Department of Neurology, Fachklinik Enzensberg, Höhenstraße 56, 87629 Hopfen am See, Germany; 2 Department of Research, Development and Quality Assurance, Klinikgruppe Enzensberg, 87629 Hopfen am See, Germany

**Keywords:** meningioma, neurorehabilitation, neuropsychology, psychology, rehabilitation, outcome

## Abstract

**Highlights:**

**What are the main findings?**

- Our retrospective analysis of a larger patient group following acute treatment of cere-bral meningioma demonstrates that neuropsychological deficits are common in these patients. The most prevalent were attention deficits, followed by executive function impairments and memory deficits.
- No correlation was identified between the extent of resection or the occurrence of complications during surgery and cognitive deficits. A prevailing trend indicated that higher-grade tumors exhibited a heightened propensity to induce cognitive impair-ment. The location of the tumor did not correlate with the impaired cognitive do-mains. At the time of discharge, a reduced number of patients demonstrated attention deficits, and those who did so exhibited symptoms of lesser severity.

**What are the implications of the main findings?**

- Due to the fact that even mild cognitive impairments can lead to problems in everyday life, we recommend detailed, standardized neuropsychological testing and, if necessary, therapy for all patients after acute treatment.
- Further studies addressing this topic are also desirable, including therapeutic studies for existing deficits.

**Abstract:**

Background: Meningiomas (MG) are the most common form of benign intracranial tumors. Neuropsychological deficits are often noticed preoperatively. After surgical removal, both improvements and persistent neuropsychological deficits have been reported. Here we present the neuropsychological characteristics of a larger patient group following acute treatment for meningioma. Methods: This retrospective study is part of an overall project investigating the postoperative characteristics and rehabilitation outcomes of 151 patients following surgical removal of MG. Patients were recruited at the neurological department of m&i-Fachklinik Enzensberg between 2019 and 2024. In addition to demographic data and tumor characteristics, the neuropsychological reports were evaluated by two experienced (neuro)psychologists. Results: 69 patients underwent standardized testing in the neuropsychology department and were thus included in the analysis. Upon admission, 52.2% of these patients exhibited attention deficits, 48% showed executive deficits, and 44% had memory impairments. No correlation was found between the extent of resection or the occurrence of complications during surgery and cognitive deficits. However, there was a trend showing that higher-grade tumors were more likely to cause cognitive impairment. The location of the tumor did not correlate with the impaired cognitive domains. At discharge, fewer patients exhibited attention deficits, and those that did had less severe symptoms. Conclusions: Meningiomas are considered to be easily treatable. However, our data show that neuropsychological impairments frequently occur after acute treatment, which may not be given sufficient attention in practice. Even mild cognitive impairments can lead to problems in everyday life or at work. We therefore recommend detailed neuropsychological diagnosis and, if necessary, therapy for all patients after acute treatment.

## 1. Introduction

Meningiomas (MG) represent the most prevalent form of intracranial tumors, exhibiting a higher incidence in female patients and an increasing prevalence with age [1,2]. As Nassiri et al. [3] and Magill et al. [4] note, the most common symptoms reported at the time of diagnosis are headache, fatigue, vision problems, seizures, sensory or motor deficits, or cognitive impairment. In cases where the MG is an incidental finding and does not present any complications, close monitoring is typically conducted through the utilization of neuroimaging techniques. However, should the MG present with any symptoms or demonstrate growth, primary treatment is surgical resection of the tumor. Depending on the grade and extent of the tumor, the use of radiosurgery or radiation therapy may be considered [5]. Preoperatively, MG frequently results in cognitive deficits ranging from small to large effect sizes, particularly in areas of attention, memory and executive functions [6,7,8]. In certain cases, the functions of memory appear to be unaffected [9]. As posited by EANO [5], the occurrence of preoperative neuropsychological deficits can be attributed to a number of factors. Firstly, the localization of the tumor itself can be considered a contributing factor. Secondly, increased intracranial pressure induced by the tumor or tumor-related edema can also be identified as a possible cause. Post-surgically, neuropsychological deficits are known to decrease, yet patients frequently exhibit ongoing cognitive impairments. However, the domains affected differ across studies. Following the removal of frontal MG, deficits in attention and executive function were reported, with memory and visuoconstructive functions being spared [9]. Three [8] and twelve months [6] after surgery still a large part of MG patients show cognitive deficits regarding attention, memory, and executive functions. There is evidence of impaired verbal memory and executive functions at least one year after tumor treatment [10]. These impairments may be attributable to antiepileptic medication [10].

The aim of the present retrospective study was to analyze the postoperative neuropsychological data of 69 patients receiving neurological rehabilitation following MG surgery or radiotherapy.

## 2. Methods

This retrospective study is part of an overall project investigating the characteristics and rehabilitation outcomes of 151 patients following surgical removal or radiotherapy of a meningioma. Two manuscripts have already been published from the overall project [11,12].

In this paper we present the neuropsychological data. Patients were recruited at the neurological department of m&i-Fachklinik Enzensberg between 2019 and 2024 using the clinic’s information system.

The evaluation was conducted in accordance with the requirements of the Declaration of Helsinki, as far as they apply to secondary analysis. All patients provided written consent at the start of rehabilitation allowing their data to be used for scientific analysis. The m&i-Fachklinik Enzensberg is a highly specialized clinic that conducts neurological rehabilitation and early rehabilitation in a multidisciplinary setting including (neuro)psychological therapy.

### 2.1. Demographic and Tumor Data

The medical records of the identified patients were used to determine routine data, including sex, age, tumor lateralization and location, tumor grade, extent of resection, occurrence of complications, number of secondary diagnoses, Barthel index (BI) [13] and length of stay.

### 2.2. Neuropsychological Data

Existing neuropsychological data were taken from the medical records of identified patients. We included all patients in the statistical analysis who underwent standardized neuropsychological testing. No patients were excluded. A list of the neuropsychological tests used can be found in the Appendix A. The patients were examined upon admission and prior to discharge from rehabilitation.

The neuropsychological reports were evaluated independently by two experienced neuropsychologists (PG and SA). We referred to the standardized data reported in the medical records, namely T-scores, percentiles, or z-scores. As the types of neuropsychological tests varied greatly from patient to patient (see Appendix A), the data were divided into three domains: (1) attention, (2) memory and (3) executive function. The results were classified as impaired if at least one parameter for a domain fell below the mean by at least one standard deviation in accordance with standard practice. If all parameters for a domain were within or above the average range, the result was classified as not impaired.

### 2.3. Interventions

If neuropsychological deficits were identified, a personalized neuropsychological training program was developed for the patient individually. Our neuropsychological training is based on the three pillars of neuropsychology: restitution, compensation, and integrative approaches. For patients requiring neuropsychological treatment, the following neuropsychological interventions were administered:

- computer-assisted restitution training
- pen-and-paper tasks in individual or group settings
- teaching memory strategies such as the method of loci
- mental imagery or storytelling techniques
- teaching compensation strategies such as note-taking, using a cell phone (e.g., calendar function)
- teaching pacing strategies
- counseling for family members.

Intervention Frequency: Attention training took place daily for 30 min whenever possible. Cognitive groups, such as the memory group, met twice a week for 60 min per session, while psychoeducational groups, such as stress management or coping with illness, met once a week for 60 min. Individual sessions took place approximately 1–2 times a week for 30–60 min. Additionally, depending on the patients’ limitations, further therapies were provided in the departments of physical therapy, occupational therapy, speech therapy, and massage departments therapy.

### 2.4. Statistical Analysis

Statistical analysis was conducted using IBM SPSS Statistics version 29. Descriptive statistics (median, mean, standard deviation (SD) and percentage) and non-parametric tests (Fisher’s exact test, chi-square test and Wilcoxon test) were used for the analysis. Missing data was not replaced. The threshold for statistical significance was set at *p* < 0.05.

## 3. Results

Between 2019 and 2024, a total of 151 patients with MG were treated at the clinic. Detailed neuropsychological examinations were performed on 69 patients.

### 3.1. Clinical Features

Table 1 shows the demographic and tumor characteristics of the 69 patients examined in the neuropsychology department.

Our findings demonstrate a gender disparity, with a higher prevalence of cases affecting women than men. The majority of tumors (56, or 81%) were classified as WHO grade I, while the remaining 13 (19%) were classified as WHO grade II. Surgical intervention was performed in 67 cases. Of these, 58 patients had the tumor removed in its entirety, while the remaining 9 patients only had partial tumor removal. Two patients received radiotherapy. Postoperative complications were observed in nine patients. Upon admission, 57 (82.6%) patients were completely independent in terms of activities of daily living (BI 70–100), 9 (13%) required some assistance (BI 30–65) and 2 (2.9%) were severely affected (BI < 30). Median BI at admission was 100 with a range from 5–100, mean BI was 87.25. Mean length of hospital stay was 32.2 days, ranging from 8 to 92 days.

### 3.2. Neuropsychological Data

Sixty-nine patients underwent standardised neuropsychological testing upon admission. Not all patients were assessed in all cognitive domains. The selection of neuropsychological tests was based upon clinical indication and the specific condition of the patient. Consequently, the number of assessed patients varies across the domains.

At admission, attentional capacities were assessed in 67 out of 69, memory function in 50 out of 69, and executive functioning in 25 out of 69 patients. Upon discharge, fewer patients were reassessed (attention: 29, memory: 7 and executive functions: 8). The figures at discharge are lower because the sample only included patients who exhibited deficits at admission, participated in the training program, were motivated to undergo a follow-up examination, and were not discharged unexpectedly.

The results of the neuropsychological tests conducted at admission and discharge are summarized in Table 2. The percentages refer to the patients assessed within each domain at the respective time point.

We evaluated the correlation between cognitive deficits present at admission and tumor grade. Although Fisher’s Exact Test revealed no correlation (*p* = 0.194), the data suggest a trend indicating that higher tumor grade leads to a higher probability of cognitive impairment following treatment. However, these results must be interpreted with caution, as only 13 patients had a WHO grade II MG.

Whether the tumor could be completely removed or only partially did not have an effect on the presence of cognitive impairment (Fisher’s Exact Test, *p* = 0.461).

Furthermore, the presence of complications intraoperatively or postoperatively did not affect the likelihood of cognitive deficits (Fisher’s Exact Test, *p* = 1.000).

Statistical evaluation did not reveal a correlation between tumor location and attention (Χ^2^ = 13.846, *p* = 0.537), memory (Χ^2^ = 9.031, *p* = 0.876), or executive deficits (Χ^2^ = 12.749, *p* = 0.388). However, this null finding may be due to low statistical power.

In terms of attention the findings show significant improvements throughout the duration of rehabilitation (z = −3.419, *p* < 0.001). Unfortunately, however, in terms of memory and executive functions, only seven respectively eight patients were assessed at both time points (see Table 3). Thus, it was not possible to conduct a rigorous statistical evaluation since the statistical power is severely restricted.

## 4. Discussion

We present the neuropsychological features of patients with meningioma (MG) who were treated at our clinic between 2019 and 2024. As our data show, cognitive impairment is common following meningioma treatment. This has also been described in the literature. Even up to 12 months after surgery, a significant proportion of MG patients still exhibit cognitive deficits in attention, memory, and executive functions [6,8]. Similarly, Di Cristofori et al. [7] examined MG patients over the age of 70 before, three months and 12 months after surgery, and demonstrated that cognitive function improved progressively over time. However, even 12 months after surgery, only around a quarter of patients had made a full cognitive recovery. Dijkstra et al. [10] described impairments in executive functions and verbal memory at least one year after tumor treatment. Similarly, Tucha et al. [9] found deficits in attentional and executive functions in patients with frontal MG after surgery, with memory and visuoconstructive functions remaining intact. Self-reported cognitive problems, such as headaches, behavioral changes and fatigue, were reported even five years after treatment. Interestingly, self-reported fatigue increased after treatment [3].

Our data largely corresponds with these studies, although we observed fewer cases of cognitive impairment. Upon admission, 52.2% of patients exhibited attention deficits, 44% had memory impairments and 48% showed executive deficits.

No influence of the extent of resection (complete versus incomplete) or the occurrence of complications during or after surgery on the probability of cognitive impairment was found. However, the number of patients in our sample who underwent incomplete resection or experienced intraoperative complications was small. Nevertheless, there was a trend indicating that a higher tumor grade more frequently leads to cognitive deficits. Magill et al. [4] showed that tumor grade and tumor size are directly related. The trend towards a higher probability of cognitive deficits in grade II MG in our study may be attributed to the larger tumor size. However, this is contradicted by a study by Tucha et al. [9], who found that tumor volume had no influence on cognitive functioning. Due to a lack of data, we were unable to record tumor size in our sample. Thus, further studies on this topic are necessary.

We did not find a correlation between the type of cognitive impairment (i.e., attention, memory or executive disorders) and the location of the tumor. We had expected that MG would lead to focal neuropsychological impairments. However, the group sizes were small and heterogeneous, so this finding may also be attributed to that. Bartolomei et al. [14] showed a loss of functional connectivity in brain tumor patients even in remote areas of the brain leading to diffuse changes in brain functions. This could also explain the lack of correlation. In line with our findings, Taphoorn et al. [15] found a correlation between tumor location and cognitive deficits in only 2 of 14 patients with low-grade glioma.

Data focusing on the connection between cognitive deficits and the lateralization (i.e., left versus right-sided) of the tumor are contradictory. Some studies have demonstrated a correlation between lateralization and the type of cognitive deficits in patients with MG or glioma, with left-sided tumors affecting verbal memory and right-sided tumors impairing visual-spatial abilities [10,16]. Tucha et al. [9] showed that attention function recovers differently after surgery depending on tumor localization, with frontobasal or right frontal MG showing advantages. However, these results must be treated with caution due to the small sample size. Left-sided MG may be associated with an increased risk of verbal memory impairment. Furthermore, patients with convexity MG tend to perform better than those with skull base MG [10]. However, Meskal et al. [8] found no impact of tumor location or side on cognitive function, except for complex attention, where performance after surgery was better for infratentorial tumors.

Consistent with Meskal et al., we found no correlation between lateralization and the type of cognitive deficits in our sample.

29 patients were examined at the start of rehabilitation and on discharge with regard to their attention performance. We found an improvement in attention deficits, with only just under 60% of patients affected on discharge, compared to 75% at admission. The severity of attention deficits also decreased. The retrospective study design does not allow for statements about the reason for the improvements; training effects through PC-supported training are possible, but spontaneous remission may also be possible.

The present study has many limitations related to the retrospective collection of data from a clinical sample. Cognitive performance was not assessed prior to surgery. Therefore, it is not possible to determine whether the cognitive deficits after treatment are related to the tumor itself, the treatment of the tumor, or whether treatment has impacted the severity of cognitive impairment. There is also a lot of missing data concerning cognitive performance. However, the neuropsychological data were recorded as part of the clinical treatment, providing a comprehensive overview of the cognitive performance of all MG patients admitted.

One downside is that various test procedures have been used to record attention performance, such as computer-based and paper-pencil tasks. This makes it difficult to compare the data. For future research, it would be useful to assess cognitive performance using the same test procedures for all patients to ensure comparability. However, this could raise ethical questions in the context of rehabilitation, as it might result in only certain groups of MG patients being examined. It would also be desirable to record the results preoperatively and for a certain time after the end of treatment, in order to obtain more data on the long-term course following a diagnosis of MG. Patients report cognitive deficits, fatigue, headaches, weakness and behavioral changes even after five years [3]. Even 10 years after MG, self-reported health-related quality of life remains impaired, with complaints focusing on cognition and sleep disturbances [17]. This highlights the importance of neurological and routine neuropsychological diagnostics and treatment following surgical treatment and radiotherapy for MG. Further studies addressing this topic are also desirable, including therapeutic studies for existing deficits. In addition, incorporating neurobiological factors such as neuroinflammation, oxidative stress, or neurotransmitter imbalance would be an interesting avenue for further research.

## 5. Conclusions

Approximately 50% of patients referred to neurological rehabilitation following acute treatment for MG exhibited cognitive impairments in the areas of attention, memory, and executive function. The findings show a significant improvement of attentional capacities throughout the duration of rehabilitation. In respect to memory and executive functions, there was insufficient data to perform a rigorous statistical analysis. This emphasizes the need for routine cognitive assessment following MG. In future studies, it would be desirable to assess preoperative cognitive status, conduct long-term follow-up preferably for several years, and evaluate therapeutic strategies.

## Figures and Tables

**Table 1:** Demographic and tumor characteristics of neuropsychological MG patients.

Characteristics	Neuropsychological MG Patients (*n* = 69)
Sex	
Male	20 (29%)
Female	49 (71%)
Age (years)	56.6 (23–84)
Tumor lateralization	
Right-sided	29 (42%)
Left-sided	37 (54%)
Bilateral	3 (4%)
Tumor localization	
Frontal	30 (44%)
Temporal	11 (16%)
Parietal	14 (20%)
Occipital	5 (7%)
Sphenoid wing	10 (11%)
Cerebellar	2 (2%)
Others	9 (13%)
Tumor grade (WHO)	
I	56 (81%)
II	13 (19%)
Resection	
Complete	58 (84%)
Incomplete	9 (13%)
Radiotherapy	2 (3%)
Complication during or after surgery	
Yes	9 (13%)
No	58 (84%)
Not applicable (Radiotherapy)	2 (3%)
Secondary diagnoses (number)	5.6 (1–14)
BI (median)	100 (5–100)
BI (mean)	87.25
BI 70–100	57 (82.6%)
BI 30–65	9 (13%)
BI < 30	2 (2.9%)
Length of stay (days, mean)	32.2 (8–92)

**Table 2:** Number of deficits in attention, memory, and executive functions in neuropsychologically examined MG patients at the time of admission to and discharge from rehabilitation.

	At Admission n (%)	At Discharge n (%)
Attention	67 (100)	29 (100)
No deficits	32 (47.8)	12 (41.4)
Deficits	35 (52.2)	17 (58.6)
Memory	50 (100)	7 (100)
No deficits	28 (56)	3 (42.9)
Deficits	22 (44)	4 (57.1)
Executive functions	25 (100)	8 (100)
No deficits	13 (52)	5 (62.5)
Deficits	12 (48)	3 (37.5)

**Table 3:** Attention performance during the rehabilitation in 29 Patients.

	At Admission (%)	At Discharge (%)
No deficits	7 (24.1)	12 (41.4)
Deficits	22 (75.9)	17 (58.6)

## Data Availability

The datasets generated during and/or analyzed during the current study are available from the corresponding author on reasonable request. The data are not publicly available due to privacy reasons.

## Abbreviations

BI	Barthel Index
MG	Meningioma

## Appendix A

The neuropsychological tests used were:

Attention:
Testbatterie zur Aufmerksamkeitsprüfung (TAP), Version 2.1. Fimm B, Zimmermann P. 2nd ed. Herzogenrath: Psytest; 2008
Testbatterie zur Aufmerksamkeitsprüfung (Version Mobilität) Version 1.3.1. Zimmermann P, Fimm B. 1st ed. Freiburg: Psytest; 2017
Nürnberger-Alters-Inventar. Oswald WD, Fleischmann UM. 3rd ed. Göttingen: Hogrefe; 1995

Memory:
Verbaler Lern- und Merkfähigkeitstest. Helmstaedter C, Lendt M, Lux S. 1st ed. Göttingen: Hogrefe; 2001
NAB Modul Gedächtnis. Petermann F, Jäncke, L Waldmann HC. 1st ed. Bern: Hogrefe; 2016
Rey Complex Figure Test and Recognition Trial. Meyers JE, Meyers KR. 1st ed. Lutz: Psychological Assessment Resources; 1995

Executive Function:
Turm von London—Deutsche Version. Tucha O, Lange KW. 1st ed. Göttingen: Hogrefe; 2004
Regensburger Wortflüssigkeits-Test. Aschenbrenner S, Tucha O, Lange KW. 1st ed. Göttingen: Hogrefe; 2000
HOTAP—Handlungsorganisation und Tagesplanung. Menzel-Begemann. 1st ed. Göttingen: Hogrefe; 2010

## References

[1] Wiemels J., Wrensch M., Claus E.B. (2010). Epidemiology and etiology of meningioma. J. Neuro-Oncol..

[2] Claus E.B., Bondy M.L., Schildkraut J.M., Wiemels J.L., Wrensch M., Black P.M. (2005). Epidemiology of intracranial meningioma. Neurosurgery.

[3] Nassiri F., Suppiah S., Wang J.Z., Badhiwala J.H., Juraschka K., Meng Y., Nejad R., Au K., Willmarth N.E., Cusimano M. (2020). How to live with a meningioma: Experiences, symptoms, and challenges reported by patients. Neuro-Oncol. Adv..

[4] Magill S.T., Young J.S., Chae R., Aghi M.K., Theodosopoulos P.V., McDermott M.W. (2018). Relationship between tumor location, size, and WHO grade in meningioma. Neurosurg. Focus.

[5] Goldbrunner R., Stavrinou P., Jenkinson M.D., Sahm F., Mawrin C., Weber D.C., Preusser M., Minniti G., Lund-Johansen M., Lefranc F. (2021). EANO guideline on the diagnosis and management of meningiomas. Neuro-Oncol..

[6] Rijnen S.J.M., Meskal I., Bakker M., De Baene W., Rutten G.M., Gehring K., Sitskoorn M.M. (2019). Cognitive outcomes in meningioma patients undergoing surgery: Individual changes over time and predictors of late cognitive functioning. Neuro-Oncol..

[7] Di Cristofori A., Zarino B., Bertani G., Locatelli M., Rampini P., Carrabba G., Caroli M. (2018). Surgery in elderly patients with intracranial meningioma: Neuropsychological functioning during a long term follow-up. J. Neuro-Oncol..

[8] Meskal I., Gehring K., van der Linden S.D., Rutten G.J., Sitskoorn M.M. (2015). Cognitive improvement in meningioma patients after surgery: Clinical relevance of computerized testing. J. Neuro-Oncol..

[9] Tucha O., Smely C., Preier M., Becker G., Paul G.M., Lange K.W. (2003). Preoperative and postoperative cognitive functioning in patients with frontal meningiomas. J. Neurosurg..

[10] Dijkstra M., van Nieuwenhuizen D., Stalpers L.J., Wumkes M., Waagemans M., Vandertop W.P., Heimans J.J., Leenstra S., Dirven C.M., Reijneveld J.C. (2009). Late neurocognitive sequelae in patients with WHO grade I meningioma. J. Neurol. Neurosurg. Psychiatry.

[11] Gdynia H.J., Schneiderat P., Gratzer A., Wiedhopf K., Gdynia N., Haase I. (2024). Clinical features and rehabilitation outcome after surgical treatment of spinal meningioma. Spinal Cord Ser. Cases.

[12] Gdynia N., Haase I., Gratzer A., Auer S., Gdynia H.-J. (2026). Team-Based Long-Term Multidisciplinary Inpatient Neurological Rehabilitation After Surgery of Cerebral Meningioma—Outcome and Confounding Factors. Brain Sci..

[13] Lübke N., Meinck M., Von Renteln-Kruse W. (2004). The Barthel Index in geriatrics. A context analysis for the Hamburg Classification Manual. Z. Gerontol. Geriatr..

[14] Bartolomei F., Bosma I., Klein M., Baayen J.C., Reijneveld J.C., Postma T.J., Heimans J.J., van Dijk B.W., de Munck J.C., de Jongh A. (2006). How do brain tumors alter functional connectivity? A magnetoencephalography study. Ann. Neurol..

[15] Taphoorn M.J., Heimans J.J., Snoek F.J., Lindeboom J., Oosterink B., Wolbers J.G., Karim A.B. (1992). Assessment of quality of life in patients treated for low-grade glioma: A preliminary report. J. Neurol. Neurosurg. Psychiatry.

[16] Scheibel R.S., Meyers C.A., Levin V.A. (1996). Cognitive dysfunction following surgery for intracerebral glioma: Influence of histopathology, lesion location, and treatment. J. Neuro-Oncol..

[17] Nassiri F., Price B., Shehab A., Au K., Cusimano M.D., Jenkinson M.D., Jungk C., Mansouri A., Santarius T., Suppiah S. (2019). Life after surgical resection of a meningioma: A prospective cross-sectional study evaluating health-related quality of life. Neuro-Oncol..

